# The Study of Association Between Polymorphism of TNF-α Gene’s Promoter Region and Recurrent Pregnancy Loss

**Published:** 2018

**Authors:** Roshanak Aboutorabi, Ehsan Behzadi, Mohammad Javad Sadegh, Seyed Pooriya Fatehi, Soroosh Semsarzadeh, Yasaman Zarrin, Mohammad Kazemi, Laleh Rafiee, Fatemeh Sadat Mostafavi

**Affiliations:** 1- Infertility Laboratory, Beheshti Hospital, School of Medicine, Isfahan University of Medical Sciences, Isfahan, Iran; 2- Department of Anatomical Sciences and Molecular Biology, School of Medicine, Isfahan University of Medical Sciences, Isfahan, Iran; 3- School of Medicine, Isfahan University of Medical Sciences, Isfahan, Iran; 4- Department of Genetics, School of Medicine, Isfahan University of Medical Sciences, Isfahan, Iran; 5- Applied Physiology Research Center, Isfahan University of Medical Sciences, Isfahan, Iran

**Keywords:** Polymorphism, Recurrent pregnancy loss, Tumor necrosis factor alpha

## Abstract

**Background:**

According to the literature review, polymorphisms of tumor necrosis factor alpha’s (TNF-α) promoter region are probably the genetic risk factors of recurrent pregnancy loss. This study has investigated five single nucleotide polymorphisms in the TNF-α gene’s promoter region to evaluate their relationship with recurrent pregnancy loss disorder.

**Methods:**

Blood samples were taken from 65 women with recurrent pregnancy loss (Case group) and 65 healthy women with a history of successful pregnancy (Control group). Polymerase chain reaction with high resolution melting (PCR-HRM) analysis was done to determine the promoter region of -308G/A, -850T/C, -238G/A, -1031T/C and -863A/C TNF-α polymorphisms. The data were assessed using logistic regression models. P values less than 0.05 were considered statistically significant.

**Results:**

Significant associations were found between recurrent pregnancy loss and -863C/A (p=0.000), -308G/A (p=0.045), and -238G/A (p=0.034) polymorphisms. TNF-α polymorphisms of -863C and -238G may be susceptible factors of recurrent pregnancy loss cases. The -308G polymorphism has an important role in maintaining pregnancy.

**Conclusion:**

The -863C/A and -238G/A TNF-α polymorphisms are possible genetic risk factors of recurrent pregnancy loss and might be its predictive markers.

## Introduction

Pregnancy loss is a frequent problem among women and occurs approximately in one eighth of clinically recognized pregnancies, most often within three months of conception (1, 2). Recurrent pregnancy loss definition, as described by the European Society for Human Reproduction and Embryology (ESHRE) is three or more repeated pregnancy losses happening before the 20th week of gestation (3, 4). However, other societies, such as American Society for Reproductive Medicine (ASRM), define recurrent miscarriage as two or more pregnancy losses (5–7). The likelihood of pregnancy loss is 5% higher for women who have suffered a miscarriage during their first pregnancy than for those who have not (8).

Several factors such as age, lifestyle, sperm quality, uterine anomalies, hormonal and metabolic disorders, infectious diseases, and genetic causes could be related to recurrent pregnancy loss (6, 9). Some studies have found a relationship between recurrent pregnancy loss and various genetic polymorphisms in promoter region of tumor necrosis factor alpha (TNF-α) gene (10).

The balance in secretory cytokines from T-helper cells (Th) has an important role in immune system disorders, blood coagulation, angiogenesis, and cell death. Also, it is associated with maintaining pregnancy (11–13). Several alterations in cytokines have been reported in recurrent pregnancy loss. For example, over-expression of cytokines secreted from Th1 seems to be related to cell-mediated immunity down-regulation and is correlated with recurrent pregnancy loss (14–18). These alterations can have a deleterious effect on pregnancy (19). Predominance of Th2-type immunity explains maternal tolerance toward fetal antigens, which protects the fetus from maternal Th1 attack (20). This means that Th2 cytokines are associated with successful pregnancy (19, 21).

Among these cytokines, TNF-α is a multifunctional pro-inflammatory cytokine which may affect many regulatory mechanisms specially coagulation and endothelial functions (14, 22). For example, scientists found association between the TNF-α -308G/A gene polymorphism and the risk of Ischemic Heart Diseases. However, the role of TNF-α -238G/A, -857C/T, -863C/A, -1031T/C and other TNF-α gene polymorphisms are in doubt (23). Previous studies have sought the genes responsible for recurrent pregnancy loss pathogenesis (11, 24–26). TNF-α single nucleotide polymorphisms have been associated with altered TNF-α secretion. It is hypothesized that they have a role in pregnancy complications such as recurrent pregnancy loss (27–29).

TNF-α is a human leukocyte antigen system gene which is located on the short arm of chromosome 6p21 (30). TNF-α gene expression is regulated at the transcriptional level. Polymorphisms in the promoter region can influence TNF-α gene’s expression (31). Many studies have tried to explain the association between various single nucleotide polymorphisms and recurrent pregnancy loss prevalence. These studies have obtained different results. TNF-α polymorphisms have been associated with a variety of disorders related to fertility, such as endometriosis and recurrent pregnancy loss (32).

This study has investigated five single nucleotide polymorphisms in TNF-α gene’s promoter region to evaluate their relationship with recurrent pregnancy loss disorder. This report has shown the role of single nucleotide polymorphisms in TNF-α gene’s promoter region to define risk factors of recurrent pregnancy loss in Isfahan city, Iran.

## Methods

Blood samples were collected from 65 women with recurrent pregnancy losses (Mean age of 29.75±4.92 years old) and 65 healthy women who had a history of successful pregnancy (Mean age of 29.04±7.25 years old). The recurrent pregnancy loss patients (case group) were enrolled between September 2014 and June 2015 in Shahid Beheshti hospital in Isfahan city, Iran. The healthy women (Control group) were also recruited from the same hospital based on the following criteria: 1) being pregnant, 2) having regular menstrual cycles, 3) a history of at least one natural delivery, 4) no history of pregnancy loss, and 5) karyotype 46, XX. This study was conducted in accordance with the ethical protocols of the Isfahan University of Medical Science’s Research Committee. All participants were informed of the study protocol and completed a consent form before participating in the study. All patients were diagnosed with recurrent pregnancy loss if they had a history of at least two consecutive spontaneous abortions before 20 weeks of gestation. No participant had a history of smoking or alcohol use.

### Genotyping:

In this case-control study, 4 *ml* of peripheral blood was collected from 65 women with recurrent pregnancy loss and 65 women who were pregnant and had at least one normal delivery before their participation. Genomic DNA samples were extracted from peripheral blood using the Genomic DNA Extraction Kit (Genetbio, South Korea) according to the manufacturer’s instruction. Five single nucleotide polymorphisms, *i.e*. rs361525, rs1800630, rs1799724, rs1799964 and rs1800629, were identified by the National Center for Biotechnology Information data bank, and primers were designed by Primer3plus software (Table 1).

**Table 1.** The primer sequences of TNF-α gene’s promoter polymorphisms

**SNP**	**Primer name**	**Sequence**	**Amplicon size (*bp*)**
rs361525	-238-F	ACCTGGTCCCCAAAAGAAAT	157
	-238-R	GCATCAAGGATACCCCTCAC	
rs1800630	-863-F	GGAGAATGTCCAGGGCTATG	134
	-863-R	TCTTCTTAAACGTCCCCTGTATTC	
rs1799724	-850-F	CAGGAGACCTCTGGGGAGAT	137
	-850-R	TCCTGGAGGCTCTTTCACTC	
rs1799964	-1031-F	TCAGGGATATGTGATGGACTCA	179
	-1031-R	TCCCCAGAGGTCTCCTGTAA	
rs1800629	-308-F	GCCCCTCCCAGTTCTAGTTC	170
	-308-R	CTTCTGGGCCACTGACTGAT	

Real-time polymerase chain reaction (PCR) with high-resolution melting (HRM) analysis was applied for the genotype analysis using Corbett Rotor-Gene 6000 real-time rotary analyzer (Corbett, Australia). The final reaction mixture contained Type-it HRM Master Mix (QIAGEN) with inter-calating DNA-binding Eva green dye, primer mix (0.7 *μM* of each primer), genomic DNA (50 *ng*), and RNase-Free Water in a total volume of 20 *μl*. PCR reactions were performed in triplicate.

The PCR program is made up of an initial denaturation-activation step at 95*°C* for five minutes, continued by a 45-cycle program (Denaturation at 95*°C* for 10 *s*, annealing and extension at 60*°C* for 30 *s*; an HRM step from 60*°C* to 95*°C* rising 0.2*°C* per second).

HRM data were analyzed using the Rotor-Gene Q software supplied with the instrument. Sequence variations were distinguished from wild-type samples by different shapes of normalized and temperature-shifted melting curves. Then, the results of HRM were confirmed again by the PCR with restriction fragment length polymorphism technique in 10% of samples randomly and by direct sequencing in six samples.

### Statistical analysis:

Departure from the Hardy-Weinberg equilibrium for the five single nucleotide polymorphisms was tested with an exact test. Single nucleotide polymorphisms’ differences in TNF-α gene’s promoter region were assessed using logistic regression models. Odds ratio and 95% confidence intervals were used to examine the association between TNF-α polymorphisms and recurrent pregnancy loss risk. The data were presented as mean±standard deviation (Continuous variables) or percentages (Categorical variables). Statistical analysis was done using statistical package for the social sciences (SPSS) version 22 (Chicago, Illinois, USA). The p-values less than 0.05 were considered statistically significant.

## Results

The average gestational age was 8.24±2.50 weeks, and number of miscarriages per person was 2.55±0.70 (Table 1). No significant difference was observed between the mean age of case and control groups (Table 2).

**Table 2.** Descriptive characteristics of case group (65 women)

**Characteristic**	**Case group**
Age (Years)	29.75±4.92
Previous pregnancy loss (Mean±SD)	2.55±0.70
Recurrent pregnancy loss < 14 weeks (%)	97.05%
Total mean gestational age (Weeks)	8.24±2.50 (169 abortions)
First mean gestational age (Weeks)	7.66±1.77 (65 cases)
Second mean gestational age (Weeks)	7.94±1.98 (65 cases)
Third mean gestational age (Weeks)	9.18±3.19 (31 cases)
Fourth mean gestational age (Weeks)	12.33±4.80 (8 cases)

**Figure 1.**
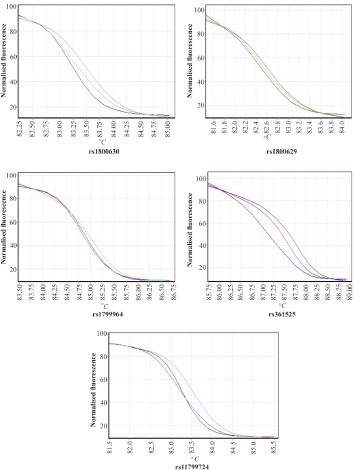
Normalized graphs for HRM of different TNF-α gene’s promoter polymorphisms

Recurrent pregnancy loss is defined as loss of gestation in less than 20 weeks of pregnancy. 97.05% of the embryos in our case group had been lost up to 14 weeks of gestation (Table 2). Total mean of gestational age was 8.24±2.50 weeks in 169 abortions in up to four gestations for each woman. The mean±standard deviation of the first, second, third and fourth gestational ages were 7.66±1.77 (65 cases), 7.94±1.98 (65 cases), 9.18±3.19 (31 cases) and 12.33±4.80 (Eight cases), respectively. Figure 1 illustrates the melting curve for each SNP.

Genotype frequencies of 5 SNPs in controls did not show any significant deviations from Hardy–Weinberg equilibrium in the controls. Polymorphisms in TNF-α gene’s promoter region were assessed in five sites (Table 3). The results revealed that -863C/A (p<0.001), -308G/A (p= 0.045) and -238G/A (p=0.034) polymorphisms were significantly related to recurrent pregnancy loss. But -1031T/C (p=0.172) and -857C/T (p=0.108) polymorphisms had no association with recurrent pregnancy loss.

**Table 3.** Genotype and allele analysis of the -863C/A, -857C/T, 308G/A, 1031C/T and 238G/A TNF-α polymorphisms in case and control groups

**Genotype**	**Control group**	**Case group**	**Odds ratio (95% confidence interval)**	**p**	**Power**	**Genotype Significant**
**TNF-α-863C/A (rs1800630)**						
CA	15 (23%)	6 (9%)	0.185 (0.068–0.506)	0.001	0.59	
AA	26 (40%)	1 (1.5%)	0.031 (0.007–0.142)	<0.001	0.99	<0.001
CC	24 (37%)	58 (89%)	1.000 (reference)		0.99	
Dominant (CC vs. CA+AA)			12.390 (5.093–30.143)	<0.001		
Recessive (CC+CA vs. AA)			21.937 (4.947–97.275)	<0.001		
**TNF-α-857C/T (rs1799724)**						
CC	46 (70%)	35 (53%)	0.109 (0.005–2.180)	0.147	0.51	
CT	19 (29%)	27 (41%)	0.201 (0.010–4.126)	0.298	0.30	0.108
TT	0 (0%)	3 (4.6%)	1.000 (reference)		-	
Dominant (CC vs. CT+TT)			0.494 (0.243–1.005)	0.052		
Recessive (CC+CT vs. TT)			0.239 (0.026–2.194)	0.206		
**TNF-α-308G/A (rs1800629)**						
GG	55 (85)	45 (69%)	2.736 (1.165–7.743)	0.039	0.58	
GA	10 (15)	12 (18%)	1.452 (.585–3.605)	0.421	0.074	0.045
AA	0 (0)	8 (12%)	1.000 (reference)		-	
Dominant (GG vs. GA+AA)			0.033 (0.004–0.256)	0.001		
Recessive (GG+GA vs. AA)			0.098 (0.012–0.794)	0.030		
**TNF-α-1031C/T (rs1799964)**						
TT	0 (0%)	41 (63%)	0.066 (0.004–1.204)	0.067	-	
CT	17 (26%)	18 (27%)	0.081 (0.004–1.553)	0.095	0.052	0.172
CC	48 (73%)	6 (9%)	1.000 (reference)		1	
Dominant (CC vs. CT+TT)			0.617 (0.297–1.284)	0.197		
Recessive (CC+CT vs. TT)			0.128 (0.015–1.069)	0.058		
**TNF-α-238G/A (rs361525)**						
GA	22 (33%)	15 (23%)	0.505 (0.235–1.085)	0.080	0.24	
AA	7 (10%)	0 (0%)	0.091 (0.011–.757)	0.027	-	0.034
GG	36 (55%)	50 (76%)	1.000 (reference)		0.72	
Dominant (GG vs. GA+AA)			2.585 (1.233–5.416)	0.012		
Recessive (GG+GA vs. AA)			8.949 (1.087–73.690)	0.042		

Frequencies of alleles in each of the polymorphisms were evaluated by odds ratio and 95% confidence interval. There was a strong significant association between TNF-α -863C polymorphism occurrence (p<0.001) and recurrent pregnancy loss. Also, a significant association was observed between -238G TNF-α polymorphism occurrence (p=0.012) and the case group. This relationship could express increasing chances of recurrent pregnancy loss.

On the other hand, -308G TNF-α polymorphisms significantly decreased odds of recurrent pregnancy loss, showing a protective effect. In analysis of the alleles, p-value in -857C/T polymorphism, CC *vs*. CT+TT (p=0.052) and also CC+CT *vs*. TT in -1031C/T polymorphism (p=0.058) were very close to become significant.

## Discussion

The exact mechanism underlying recurrent pregnancy loss is not known. Some environmental risk factors, genetic and immunologic causes, and chromosomal abnormalities are known to be responsible (33). TNF-α is an apoptosis inducing factor in tumor cells. It has anti-tumor activity through cell cycle arrest mechanism (34–36). Also, TNF-α is a primary inflammatory cytokine which has an essential role in keeping the balance of body mechanisms such as coagulation, angiogenesis and endothelial functions (37).

Our study tried to demonstrate the predisposing effect of some TNF-α single nucleotide polymorphisms related to recurrent pregnancy loss. The findings showed some susceptible single nucleotide polymorphisms in recurrent pregnancy loss.

Based on our results, -863C/A polymorphism had a significant relationship with recurrent pregnancy loss. In this single nucleotide polymorphism, C allele caused tendency to abortion. Similarly, in a study by Piosik et al., it was revealed that -863C polymorphism is associated with higher level of TNF-α and may cause abortion (38). However, Jang et al. in 2016 found a significant relationship between -863C/A polymorphism and abortion, but they emphasized on A allele as a susceptible factor for this disorder (30). This controversy about the allele is due to odds ratio of our study which shows CA/CC odds ratio is 0.185 and AA/CC odds ratio is 0.031 (Table 3). These values were 2.076 and 2.846 in Jang et al.’s study, respectively. These differences can be explained in three points: 1) considering regional and geographic impression on genomics; 2) number of samples, and 3) the difference between single nucleotide polymorphisms extraction methods (Restriction fragment length polymorphism-PCR versus high-resolution melting-PCR).

It was found that the TNF-α -238G allele could be a genetic risk factor that increases the chances of abortion and TNF-α promoter activity. In this regard, a study in India concluded that G allele reverses impression on TNF-α promoter activity (10).

Polymorphisms in TNF-α gene have been extensively evaluated in recurrent pregnancy loss cases. Most studies evaluated single nucleotide polymorphism at the -308G/A, -238G/A and -863C/A polymorphisms of TNF-α gene’s promoter region (39–41).

The -308G/A polymorphism was significantly different between the two study groups. Here, the G allele had a protective effect against spontaneous abortion. In this regard, a study found a possible association between -308G/A TNF-α polymorphism and recurrent pregnancy loss too (42). However, another study revealed that this polymorphism is not associated with early recurrent pregnancy loss (43).

A study found that -1031C/T TNF-α polymorphism has no significant correlation with recurrent pregnancy loss (30). Piosik et al. arrived at similar results (38). In a study in 2010, this polymorphism had a significant correlation with recurrent pregnancy loss which was due to C allele (41).

According to our findings, -857C/T polymorphism had no relation with increasing recurrent pregnancy loss risk. Similarly, two studies, one in Korea and another in Denmark, have shown no significant correlation for this polymorphism (30).

In these two SNPs (-1031C/T and -857C/T), post hoc power analysis showed that the sample size was not adequate to detect a significant difference between the groups (Table 3).

In a similar research in our geographical region (Bahrain), Finan et al. evaluated seven single nucleotide polymorphisms in TNF-α gene’s promoter region. They found that -238G/A polymorphism was significantly different between their case and control groups (41) and frequency of G allele in -238G/A polymorphism was related to increased abortion risk which is consistent with our findings. These results were contrary to Parveen et al.’s study (10) which revealed that G allele in -238G/A polymorphism may be associated with low production of TNF-α. Subsequently, this single nucleotide polymorphism may have an important role in maintaining pregnancy (10). In another research, -238A TNF-α polymorphic variant was associated with early recurrent pregnancy loss (43).

## Conclusion

Our study reveals that TNF-α polymorphisms, in particular -863C/A and -238G/A variants, are significantly associated with idiopathic recurrent pregnancy loss and -308G allele had a protective effect against spontaneous abortion. A meta-analysis and more additional replication studies are necessary on other racial groups to evaluate and confirm the findings.
